# Chronic pain in multi-traumatized outpatients with a refugee background resettled in Norway: a cross-sectional study

**DOI:** 10.1186/s40359-015-0064-5

**Published:** 2015-03-15

**Authors:** Dinu-Stefan Teodorescu, Trond Heir, Johan Siqveland, Edvard Hauff, Tore Wentzel-Larsen, Lars Lien

**Affiliations:** 1 Department of Public Health, Hedmark University College, Elverum, Norway; 2 Norwegian Centre for Violence and Traumatic Stress Studies, Oslo, Norway; 3 R & D Department, Mental Health Services, Akershus University Hospital, Oslo, Norway; 4 Division of Mental Health and Addiction, Oslo University Hospital, Oslo, Norway; 5 Institute of Clinical Medicine, Faculty of Medicine, University of Oslo, Oslo, Norway; 6 Centre for Child and Adolescent Mental Health, Eastern and Southern Norway, Oslo, Norway; 7 Innlandet Hospital Trust, PO Box 104, N-2381 Brumunddal, Norway

**Keywords:** Chronic pain, Comorbid chronic pain and PTSD, Resettled refugees, Traumatized refugees, DESNOS

## Abstract

**Background:**

Traumatized refugees often report significant levels of chronic pain in addition to posttraumatic stress disorder symptoms, and more information is needed to understand pain in refugees exposed to traumatic events. This study aimed to assess the frequency of chronic pain among refugee psychiatric outpatients, and to compare outpatients with and without chronic pain on trauma exposure, psychiatric morbidity, and psychiatric symptom severity.

**Methods:**

We conducted a cross-sectional study of sixty-one psychiatric outpatients with a refugee background using structured clinical diagnostic interviews to assess for traumatic events [Life Events Checklist (LEC)], PTSD (Posttraumatic Stress Disorder) and complex PTSD [Structured Clinical Interview for DSM-IV PTSD Module (SCID-PTSD) and Structured Interview for Disorders of Extreme Stress (SIDES)], chronic pain (SIDES Scale VI) and psychiatric symptoms [M.I.N.I. International Neuropsychiatric Interview (M.I.N.I.)]. Self-report measures were used to assess symptoms of posttraumatic stress [Impact of Event Scale-revised (IES-R)], depression and anxiety [Hopkins Symptom Checklist (HSCL-25)] and several markers of acculturation in Norway.

**Results:**

Of the 61 outpatients included, all but one reported at least one chronic pain location, with a mean of 4.6 locations per patient. Chronic pain at clinical levels was present in 66% of the whole sample of outpatients, and in 88% of the outpatients with current PTSD diagnosis. The most prevalent chronic pain locations were head (80%), chest (74%), arms/legs (66%) and back (62%). Women had significantly more chronic pain locations than men. Comorbid PTSD and chronic pain were found in 57% of the outpatients. Significant differences were found between outpatients with and without chronic pain on posttraumatic stress, psychological distress, and DESNOS severity.

**Conclusions:**

Chronic pains are common in multi-traumatized refugees in outpatient clinics in Norway, and are positively related to symptomatology and severity of psychiatric morbidity. The presence of chronic pain, as well as comorbid chronic pain and PTSD, in psychiatric outpatients with a refugee background call for an integrated assessment and treatment for both conditions.

## Background

Many refugees have been exposed to multiple traumas (Steel et al. 2002; Elklit et al. 1998) and are at high risk for developing Posttraumatic Stress Disorder (PTSD) (Roth et al. 2006; Ai et al. 2002; Lavik et al. 1996; Ferrada-Noli et al. 1998). Among the most traumatizing exposures with a particularly high risk for later PTSD symptom development are torture and war trauma (Johnson and Thompson 2008; Jaranson et al. 2004). Besides mental health problems, somatic pain problems are also common in refugees (Gerritsen et al. 2006).

Pain in the aftermath of a traumatic event has been identified as a risk factor for the development of PTSD (Norman et al. 2008), and often PTSD and chronic pain are concomitant (Moeller and Bertram et al. 2012; Beck and Clapp 2011; Beckham et al. 1997). Patients seeking treatment for chronic pain also have higher rates of PTSD (Villano et al. 2007; Dunn et al. 2011; Sharp 2004; Andersen et al. 2012). The prevalence of pain in veterans seeking treatment for PTSD was found to be higher than the prevalence of PTSD in veterans seeking treatment for pain (Asmundson et al. 2002). This difference may be explained by the nature of traumatic events that are often associated with physical injuries, such as war injuries or torture. The severity of PTSD symptoms was found to be related to higher intensity of pain (Hermansson et al. 2001). The highest rates of chronic pain are found in torture exposed refugees (Carinci et al. 2010; Thomsen et al. 2000; Williams et al. 2010), and chronic pain in tortured refugees has been found to have a strong impact on daily functioning (Prip et al. 2011).

PTSD and chronic pain comorbidity is high in refugees, and a Norwegian study reported chronic pain prevalence of 76% in a clinical sample of refugees with PTSD symptoms (Dahl et al. 2006), a finding in line with similar research from Denmark (Carlsson et al. 2005), Great Britain (Taylor et al. 2013), and Germany (Liedl et al. 2010).

A Swedish study investigated gender differences in somatic pain and found that being a female, regardless of ethnicity (Kurdish-born or Swedish-born), resulted in higher odds for poor psychological well-being (Taloyan et al. 2008). This gender difference in psychological well-being among refugees is also consistent with a Norwegian study (Hjellset et al. 2011). The Oslo Immigrant Health study found that musculoskeletal disorders in immigrant groups were up to 8-fold higher as compared with Norwegians, with immigrant women reporting greater proportions than men (Kummar et al. 2008).

The impact of stress on both mental and physical health has been acknowledged for many years, a relationship influenced by biological, psychological, behavioral and social determinants (Schneiderman et al. 2005).

Several models have been developed in order to explain the complex interactions between pain and PTSD such as the “mutual maintenance theory” (Sharp and Harvey 2001), the “shared vulnerability model” (Asmundson et al. 2002), the “fear-avoidance model” (Norton and Asmundson 2003) and the “perpetual avoidance model” (Liedl and Knaevelsrud 2008).

We shall explain in more depth only one model which we find most relevant for our study, the mutual maintenance theory. This theory proposes that PTSD and chronic pain maintain or exacerbate each other through seven mechanisms: attentional and reasoning biases, anxiety sensitivity, reminders of the trauma, avoidance, depression and reduced activity levels, anxiety and pain perception and cognitive demand from symptoms limiting the use of adaptive strategies. Attentional biases are common in both PTSD and chronic pain, and outpatients with PTSD will more often direct their attention towards the pain sensation, which would increase the perception of pain. Anxiety sensitivity, which implies a fear of anxiety-related bodily sensations, will amplify the anxiety responses associated with exposure to trauma and the sensation of pain (Taylor 2004). Reminders of the trauma contribute to physiological arousal and other symptoms of PTSD, and the chronic pain is perceived as a reminder of the past trauma. Avoidant coping styles, including both behavioral and cognitive avoidance strategies, are employed in both PTSD and chronic pain leading to the maintenance of symptoms of deconditioning and disability in pain patients, and the maintenance of intrusive symptoms and associated arousal in PTSD patients (Waddell et al. 1993; Foa et al. 1989). Depression and reduced levels of activity are common in both chronic pain and PTSD outpatients, leading to increased levels of disability in chronic pain outpatients, and to a lack of exposure to trauma related stimuli in PTSD outpatients (Waddell et al. 1993). The anxiety and pain perception mechanism proposes that PTSD, which is characterized by anxiety, will directly influence the perception of pain (Difede et al. 1997; Defrin et al. 2008). Finally, an overload of cognitive capacity employed in reducing the perception of pain leads to a limited use of adaptive strategies for controlling the pain (Bryant et al. 2001). Thus, in the case of chronic pain, physiological arousal, cognitive catastrophizing, and behavioral avoidance components contribute to the maintenance of PTSD, while in the case of the PTSD, physiological arousal, emotional numbing and behavioral avoidance components contribute to the maintenance or aggravation of chronic pain. Evidence from research for the mutual maintenance between symptoms of PTSD and chronic pain has been found both in longitudinal and cross sectional studies (Jenewein et al. 2009; Liedl et al. 2010).

The perpetual avoidance model may further explain the mutual maintenance of the symptoms of chronic pain and PTSD, by the PTSD re-experiencing symptom triggering arousal, which generates muscle tension in the body and leads to the development of chronic pain. The experience of pain in and of itself is distressing and an avoidance strategy may be employed to cope with this pain, which may in turn trigger the re-experiencing component of PTSD, and thus the mutual maintenance of chronic pain and PTSD. This mechanism of mutual maintenance between PTSD symptoms and chronic pain has indirect support from studies in veterans with and without PTSD which report more physical symptoms in veterans with PTSD (Baker et al. 1997; O'Toole and Catts 2008).

Refugees are often exposed to multiple or complex traumas over longer periods of time and due to this can develop Complex PTSD or Disorders of Extreme Stress Otherwise Not Specified (DESNOS). Complex PTSD has been identified in traumatized refugees (Palic and Elklit 2014) and tortured refugees (Teegen and Vogt 2002), and since 1992 has been proposed as a separate diagnostic category in order to accommodate changes in six self domains [(I) alterations in affect and impulse regulation; (II) alterations in consciousness or attention; (III) alterations in self-perception; (IV) alterations in perception of the perpetrator (not required for a DESNOS diagnosis); (V) alteration in relations with others; (VI) somatization, and (VII) alterations in systems of meaning] (Herman 1992). Complex PTSD or DESNOS was not included in the DSM-V (American Psychiatric Association 2013), but the ICD-11 Beta Draft has proposed the following definition of complex post-traumatic stress disorder or DESNOS: “Complex PTSD is a disorder that arises after exposure to a stressor typically of an extreme or prolonged nature and from which escape is difficult or impossible. The disorder is characterized by the core symptoms of PTSD as well as the development of persistent and pervasive impairments in affective, self and relational functioning, including difficulties in emotion regulation, beliefs about oneself as diminished, defeated or worthless, and difficulties in sustaining relationships” (WHO 2014).

Comorbid chronic pain and PTSD in psychiatric outpatients with a refugee background resettled in Western countries have not been investigated in many studies. The few that exist are limited in that they have made use of short questionnaires for establishing a PTSD diagnosis, and employed interpreters with a limited knowledge of the questionnaires. (Dahl et al. 2006; Hermansson et al. 2002). To improve upon this previous work, the present investigation used a clinical diagnostic structured interview to assess PTSD, in addition to assessing a wide range of psychiatric diagnoses. The aims of the study were:

1. To assess the frequency of clinical levels and location of chronic pain, and comorbid PTSD and chronic pain in psychiatric outpatients with a refugee background.
2. To compare outpatients with and without chronic pain on demographic, acculturation (Norwegian language proficiency, employment, social integration in Norway and ethnic community), trauma exposures, psychiatric morbidity, and psychiatric symptom severity variables.
3. To compare outpatients with and without PTSD on chronic pain locations.

We evaluated the following hypotheses:

1. The group of patients with chronic pain will report more psychiatric morbidity and more severe symptomatology than the group without chronic pain.
2. Women will report more chronic pain at clinical levels, more locations of pain, and more comorbid PTSD and chronic pain than men.
3. Patients with a PTSD diagnosis will have more chronic pain at clinical levels than patients without PTSD.

## Method

### Participants

Participants were recruited from 17 general psychiatric outpatient clinics in South-Eastern Norway between November 1^st^ 2008 and November 1^st^ 2009. The outpatients were informed about the study by their therapists who were making referrals to the study. A total of 63 patients were asked to participate in the study, two declined the invitation, and 61 outpatients, 25 (41%) of whom were women, accepted to participate. None of the patients withdraw their consent during the study. After the referral from the outpatients’ therapists, the researcher (the first author) evaluated the inclusion criteria in the study: previous exposure to at least one traumatic event according to criteria from the DSM-IV (American Psychiatric Association 2000), having a permanent residence in Norway, and having sufficient proficiency in spoken and written Norwegian. The exclusion criteria included suffering from a serious medical condition (neurological or organic), active psychotic episode, high current suicide risk, not having a permanent residence in Norway and having insufficient knowledge of the Norwegian language. Written informed consent was obtained from all participants at the first meeting with the researcher (the first author) prior to the clinical interview, and they were compensated 200NOK (EUR 25) for their study participation, and all travel expenses were covered as well. The outpatients were interviewed in the psychiatric clinics where they used to receive treatment, and the clinical interviews lasted for on average two hours. At the clinical interview the researcher assessed the inclusion criteria by means of an anamnestic interview and during this clinical interview the degree of knowledge of the Norwegian language was assessed directly by the researcher. At the end of the clinical interview the outpatients received a stamped envelope which included the Questionnaire (a set of self-reported questionnaires) to be completed at home and posted within a week. One telephone follow-up reminder was made if the Questionnaire was not returned after one week. The study protocol was approved by the Committee for Medical Ethics of Health Region East of Norway (REK-East).

### Measures

The first author collected data from patients through a structured clinical interview, followed by the patient’s completion of a self-report Questionnaire consisting of a set of questionnaires. Five outpatients failed to return the self-report Questionnaire, and the total scores of IES-R and HSCL-25 are based on the scores of the outpatients who returned the self-report Questionnaire.

*National origin* was assessed with the question from the self-report Questionnaire” Which country is your country of origin? We created the variable “Regional origin” by grouping the countries into three categories: 1) Eastern Europe; 2) Africa + Middle East; 3) Other. The “Other” category included refugees from Asia and Latin America.

*Employment* was assessed by one question from the self-report Questionnaire “Are you employed and paid for it?” with three response alternatives: (1) “Full-time paid employment”; (2) “Part-time paid employment”; (3) “No paid employment”.

*Having friends* was measured with the question from the self-report Questionnaire: “How many good friends do you have?” (“Count those whom you can talk to in confidence and who can help you when you need help”). We created a categorical variable “Having friends” by grouping the number of friends into two categories: 1) “0” friends 2) all numbers of friends ranging from “1” friend to “11” friends.

To assess integration into Norwegian society, *Social integration in Norway* was measured by an index based on four items from the self-report Questionnaire: (1) Knowledge of the Norwegian language; (2) Reading Norwegian newspapers in the last year; (3) Visited by Norwegians in the last year; (4) Received help/support from Norwegians in the last year. Items 2 to 4 had 4 response alternatives, higher scores indicating higher acculturation. The response alternatives were (1)“Never”; (2) “Not very often”; (3) “Weekly”; (4) “Daily”. Item 1 had 5 response alternatives: (1) “Very bad”; (2) “Bad”; (3) “Medium”; (4) “Good”; (5) “Very good”. We collapsed the response alternatives (1) and (2) for Item 1 into one category. An index score was calculated by adding the scores for the four items, with a possible range from 4 to 16 and higher scores indicating higher integration.

We measured *Social integration in the ethnic community* (non-Norwegian community) in Norway with two items from the self-report Questionnaire: (1) “How often in the last year have you participated in meetings arranged by your countrymen?” and (2) “Reading newspapers in your first language in the last year”. The items had 4 response alternatives: (1) “Never”; (2) “Very seldom”; (3) “Weekly”; (4) “Daily”. An index score was calculated by adding the scores for the two items, with a possible range from 2 to 8.

We used a validated Norwegian translation of the *Life Events Checklist (LEC)* to assess exposure to potentially traumatic events (Halvorsen and Stenmark 2010). The LEC is a 17-item self-report questionnaire, but we administered it as part of the structured interview. The LEC has good temporal stability and reliability (Gray et al. 2004).

We used the *Structured Clinical Interview for DSM-IV PTSD Module* (*SCID-PTSD)* to determine a current PTSD diagnosis in the last month. The SCID-PTSD is based on the criteria from the Diagnostic and Statistical Manual of Mental Disorders, version IV (DSM-IV) (First et al. 1996). We also assessed for the worst potential traumatic event ever and each PTSD symptom in the last month.

*The Structured Interview for Disorders of Extreme Stress (SIDES)* is a clinical structured interview with 48 items and was used to assess the seven symptom domains of complex PTSD/ DESNOS (Disorders of Extreme Stress Not Otherwise Specified) (Pelcovitz et al. 1997): (I) alterations in affect and impulse regulation; (II) alterations in consciousness or attention; (III) alterations in self-perception; (IV) alterations in perception of the perpetrator (not required for a DESNOS diagnosis); (V) alteration in relations with others; (VI) somatization, and (VII) alterations in systems of meaning. SIDES has good psychometric properties, with reported kappa values of 0.8 and internal consistency of 0.96. We used a Norwegian translation of SIDES developed by the Psychological Trauma Research Group at the University of Oslo. (SIDES version 1997(2) revised in7/2003). In our study, SIDES had a Cronbach’s alpha of 0.89.

We assessed for *Chronic pain* using the *SIDES Subscale* VI b question: “I suffer from chronic pain (circle items that apply) “, with the response alternatives (1) “Yes” and (2) “No”. Chronic pain was defined as a long-lasting pain stretching over many years, and all patients indicated that their chronic pain either began after the traumatic event (“Was this true for you *after the experience?*”) or had been present for as long as they could remember (“Has this been true for you *for as long as you can remember*?”).

We assessed *Chronic pain at clinical levels* with the *SIDES Subscale VI b* question”How true has this been for you in the last month” with the following five response categories: 1) “None; not at all”, 2) “Some trouble-did not require medical attention”, 3) “Visited a doctor, more than one medicine without relief”, 4) Several doctor visits, a hospital admission, and/or invasive diagnostic tests”, and 5) “Not applicable”. The response categories 1, 2 and 5 were collapsed into the category “Chronic pain at non-clinical levels”, and response categories 3 and 4 were collapsed into the category “Chronic pain at clinical levels”.

We identified *Pain locations* as endorsements of three items from the *SIDES Scale VI*. The first item was from the *SIDES Subscale* VI a, and stated: “I have trouble with abdominal pain”. The second item was from the *SIDES Subscale* VI b, and stated “I suffer from chronic pain” with 6 response alternatives and the possibility to endorse all that applied: “in your arms and legs”, “in your back”, “in your joints”, “during urination”, “headaches”, “elsewhere”. The third item was from the *SIDES Subscale* VI c and stated “I suffer from chest pain”.

*The total number of pain locations* was constructed by summing all the individual pains identified from the three items of the *SIDES Subscales VI a, b and c*.

We used the *M.I.N.I. International Neuropsychiatric Interview 5.0.0 (M.I.N.I.)* to assess 21 Axis I DSM-IV disorders, although we did not include the modules for PTSD and anti-social personality disorder (Sheehan et al. 1998). We used a Norwegian version of the M.I.N.I. which has been validated in a sample of psychiatric patients (Mordal et al. 2010). We calculated the *Total number of current diagnoses* by adding all the assessed M.I.N.I. 5.0.0. current diagnoses.

We used a Norwegian translation of the *Impact of Event Scale-revised (IES-R)* to assess the presence and intensity of posttraumatic stress symptoms during the last week (Weiss and Marmar 1997; Heir et al. 2010). The IES-R is a 22-item self-report questionnaire, with a standard cut-off score of ≥ 33 to indicate a PTSD diagnosis (Creamer et al. 2003). In our study, the IES-R had a Cronbach’s alpha of 0.94.

We used a validated Norwegian translation of the *Hopkins Symptom Checklist (HSCL-25)* to measure depression and anxiety. The HSCL-25 is a 25-item self-report questionnaire assessing depression and anxiety during the last week (Derogatis et al. 1974). It has two subscales assessing depression and anxiety, with cut-off-scores > 1.75 for both subscales to indicate likely depression and anxiety disorders. The HSCL-25 has been validated in refugee populations and has good psychometric properties (Mollica et al. 1987; Renner and Salem 2009; Lavik et al. 1999). We calculated a Cronbach’s alpha of 0.91 for the total scale HSCL-25 in our study. The anxiety subscale of the HSCL-25 had a Cronbach’s alpha of 0.89, while the depression subscale of the HSCL-25 had a Cronbach’s alpha of 0.92.

#### Statistical procedures

We used frequencies, means and standard deviations to describe the sample on demographic and trauma variables. Exact chi-square tests were used to compare differences between outpatients with and without chronic pain on demographic, social, trauma variables, as well as gender differences in the location of pains. We also used exact chi-square tests to assess differences in the locations of chronic pain and clinical levels of chronic pain between outpatients with and without PTSD, as well as the presence of current PTSD diagnosis in outpatients with or without chronic pain at clinical levels. We used t-tests to assess differences in types of traumatic exposures, and differences in psychiatric symptom severity between outpatients with and without chronic pain. We also used t-tests to identify differences in the total number of chronic pains between outpatients with and without PTSD. We calculated a phi coefficient to estimate the strength of statistically significant differences in the chi-square analyses, and an eta squared coefficient for the t-tests. Missing data was handled by using the pairwise exclusion of cases. In order to minimize errors of multiple testing, we adjusted other p-values using the Holm correction (Aickin and Gensler 1996). Cronbach’s alpha coefficients were calculated for all scales. All tests were two-tailed and alpha was set at p = 0.05. All statistical analyses were performed on IBM SPSS Statistics 19 (IBM SPSS Statistics Inc, Armonk, New York, USA).

## Results

### Demographic, social, and trauma characteristics

There were 36 (59%) men and 25 (41%) women originated from some 21 countries from four continents. The mean age for the whole sample was 41.7 (SD = 9.6) years. The mean of total types of exposures to traumatic events was 10 (SD = 2.4) for men and 9 (SD = 3.1) for women. The mean time since the traumatic event was 17.8 years (SD = 10.2) for men, and 16.5 years (SD = 8.8) for women, and the mean length of stay in Norway was 15.8 years (SD = 6.4 years). A current PTSD diagnosis was found in 50 (82%) of the outpatients. There were no significant differences between the outpatients with and without chronic pain on demographic, social variables, and trauma exposures. Demographic and social variables are presented in Table 1.

**Table 1:** **Demographics and social variables**

	No Chronic pain* (n = 21) N (%)	Chronic pain* (n = 40) N (%)	Chi^2^/t-value	p-value	p-value§
**Gender (n = 61)**					
Women	8 (32.0)	17 (68.0)	0.110	0.790	1.000
Men	13 (36.1)	23 (63.7)			
**Marital status (n = 61)**					
Single	7 (58.3)	5 (41.7)	4.549	0.111	0.888
Married	9 (25.0)	27 (75.0)			
Divorced	5 (38.5)	8 (61.5)			
**Ethnicity (n = 61)**					
Eastern Europe	7 (31.8)	15 (68.2)	0.160	0.941	0.941
Africa + Middle East	9 (34.6)	17 (65.4)			
Other	5 (38.5)	8 (61.5)			
**Living conditions (n = 60)**					
Living alone	6 (50.0)	6 (50.0)	1.875	0.304	1.000
Living with family/others	14 (29.2)	34 (70.8)			
**Employment (n = 53)**					
Not employed	11 (34.4)	21 (65.6)	1.607	0.471	1.000
Part time employed	5 (50.0)	4 (50.0)			
Employed	3 (23.1)	10 (76.9)			
**Having friends (n = 57)**					
Having no friends	5 (22.7)	17 (77.3)	1.299	0.381	1.000
Having friends	13 (37.1)	22 (62.9)			
**Proficiency of the Norwegian language (n = 56)**					
Low	2 (40.0)	3 (60.0)	3.242	0.183	1.000
Medium	4 (18.2)	18 (81.8)			
High	12 (41.4)	17 (67.9)			
**Social integration in Norway (n = 57)**	10.9 (2.1)	9.3 (2.6)	1.526	0.021	0.252
**Social integration in ethnic community (n = 56)**	3.9 (1.3)	3.3 (1.1)	0.924	0.058	0.522
**Years in Norway (n = 56) #** Mean (SD)	17 (9.0)	16 (6.1)	0.487	0.628	1.000
**Education (n = 55)** # Mean (SD)	14 (4.5)	12 (3.3)	2.058	0.045	0.495
**Age (n = 61)** # Mean (SD)	39 (11.4)	43 (8.2)	−1.942	0.057	0.570

Exact chi-square tests were performed; (*) Chronic pain at clinical levels; (#) *T*-test; (§) p-values adjusted for multiple testing by Holm adjustment; in bold significant p-values.

### Chronic pain, traumatic experiences, and severity of psychiatric symptomatology

All except one outpatient reported chronic pain in at least one location (98%), and 4 outpatients (7%) identified eight chronic pain locations. The mean number of pain locations was 4.6 (SD = 2.1). The most prevalent chronic pain locations were head (80%), chest (74%), arms/legs (66%), back (62%), and stomach (57%).

No significant differences in specific or total number of types of traumatic exposures were found between outpatients with or without chronic pain. Frequencies and p-values are presented in Table 2.

**Table 2:** **Differences between groups on traumatic exposures**

Variables	No Chronic pain* (n = 21) N (%)	Chronic pain* (n = 40) N (%)	Chi^2^	p-value
**Traumatic experiences (LEC)**				
Natural disaster	6 (25.0)	18 (75.0)	1.557	0.275
Fire or explosion	13 (28.3)	33 (71.7)	3.150	0.117
Transportation accident	11 (27.5)	29 (72.5)	2.469	0.158
Serious accident at work or at home	9 (31.0)	20 (69.0)	0.282	0.788
Exposure to toxic substance	4 (40.0)	6 (60.0)	0.165	0.725
Physical assault	17 (32.7)	35 (67.3)	0.469	0.706
Assault with weapon	16 (34.8)	30 (65.2)	0.011	1.000
Sexual assault	7 (38.9)	11 (61.1)	0.225	0.769
Other unwanted sexual experience	5 (35.7)	9 (64.3)	0.013	1.000
Combat or exposure to a war-zone	13 (28.9)	32 (71.1)	2.330	0.220
Captivity	11 (34.4)	21 (65.6)	0.000	1.000
Life-threatening illness or injury	8 (27.8)	21 (72.4)	1.146	0.419
Severe human suffering	19 (35.2)	35 (64.8)	0.120	1.000
Sudden, violent death	13 (31.7)	28 (68.3)	0.410	0.574
Sudden, unexpected death of someone close to you	16 (31.4)	35 (68.6)	1.285	0.291
Serious injury, harm, or death you caused to someone else	2 (20.0)	8 (80.0)	1.103	0.470
Any other stressful event or experience	18 (33.3)	36 (66.7)	0.249	0.683
Total types of traumatic exposures # Mean (SD)	9 (2.6)	10 (2.8)	−1.679	0.094

Exact chi-square tests were performed; (*) Chronic pain at clinical levels; (#) *T*-test; LEC = Life Events Checklist.

Outpatients with chronic pain at clinical levels had significantly more symptoms of PTSD (M = 55.0; SD = 15.5) than outpatients without chronic pain (M = 42.8; SD = 19.1) [t (54) = −2.56, p = 0.013, adjusted p = 0.039]. The mean difference was −12.25, 95% CI:-21.84 to −2.65, and the magnitude was small (eta squared = 0.017).

Outpatients with chronic pain at clinical levels had significantly higher distress levels than those without chronic pain at clinical levels, more depressive symptoms, more anxiety symptoms, and more DESNOS symptoms. Means, t-values and p-values are presented in Table 3.

**Table 3:** **Group comparisons on psychiatric symptoms severity and number of diagnoses**

Dependent variables	No chronic pain* (n = 21)	Chronic pain* (n = 40)			
	Mean (SD)	Mean (SD)	t-value	p-value	p-value§
IES-R	42.8 (19.1)	55.0 (15.5)	−2.558	**0.013**	**0.050**
HSCL-25 total scale	2.3 (0.6)	2.9 (0.5)	−3.503	**0.001**	**0.006**
HSCL- 25- Depression scale	2.4 (0.7)	2.0 (0.6)	−3.040	**0.004**	**0.016**
HSCL- 25-Anxiety scale	2.3 (0.6)	2.8 (0.6)	−3.302	**0.002**	**0.010**
SIDES severity	35.5 (18.2)	46.5 (17.0)	−2.337	**0.023**	0.069
Number of current diagnoses	3.8 (2.6)	5.0 (2.5)	−1.901	0.078	0.078

T-tests were performed; (*) Chronic pain at clinical levels; (§) p-values adjusted for multiple testing by Holm adjustment; in bold significant p-values; IES-R = Impact of Event Scale Revised; HSCL-25 = Hopkins Symptom Checklist Scale; SIDES = Structured Interview for Disorders of Extreme Stress.

### Chronic pain and gender

Women had significantly more chronic pain locations (M = 5.28; SD = 1.9) than men (M = 4.08; SD = 2.13) [t (59) = −2.27, p = 0.027]. The mean difference was = −1.20, 95% CI:-2.25 to −0.14, and the magnitude was small (eta squared = 0.017).

There were no significant differences between men and women in prevalence of chronic pain at clinical levels [*χ*^2^ (1, n = 61) = 0.110, p = .790, phi = .04], or prevalence of comorbid PTSD and chronic pain [*χ*^2^ (1, n = 61) = 0.033, p = 1.000, phi = −0.02]. The frequency of chronic pain by gender is presented in the Table 4.

**Table 4:** **Frequency of chronic pain locations in gender**

	Men (n = 36)		Women (n = 25)		Total population (n = 61)		Chi^2^/t-value	p-value	p-value§	
Variables	N	%	N	%	N	%				
Chronic pain locations*										
Stomach pain	16	45.7	19	54.3	35	57.3	6.007	**0.019**		0.224
Chest pain	28	62.2	17	37.8	45	73.8	0.729	0.555		1.000
Arms/legs pain	21	52.5	19	47.5	40	65.6	2.040	0.181		1.000
Back pain	19	50.0	19	50.0	38	62.3	3.387	0.106		0.957
Joints pain	16	48.5	17	51.5	33	54.1	3.297	0.116		0.930
Head pain	27	55.1	22	44.9	49	80.3	1.578	0.328		1.000
Pain during urination	10	50.0	10	50.0	20	32.8	1.000	0.408		1.000
Other pain locations	10	52.6	9	47.6	19	31.1	0.465	0.579		1.000
Chronic pain at clinical levels	23	57.5	17	42.5	40	100	0.110	0.790		-
Total number of pain locations # Mean (SD)	4.08(2.13)		5.28(1.86)		4.57(2.09)		−2.271	**0.027**		-

Exact chi-square tests were performed; (*) Chronic pain at clinical levels; (#) T-tests; (§) p-values adjusted for multiple testing by Holm adjustment; in bold significant p-values.

### Chronic pain and PTSD

Seventy percent of the outpatients with chronic pain at clinical levels had also a current PTSD diagnosis, but no significant difference between the groups with and without chronic pain in having a current PTSD diagnosis was found [*χ*^2^ (1, n = 61) = 1.44, p = 0.164, phi = 0.20].

Further, 88% of the outpatients with current PTSD diagnosis had chronic pain at clinical levels, but no significant difference between the groups with and without current PTSD in having a chronic pain at clinical levels was found [*χ*^2^ (1, n = 61) = 1.44, p = 0.164, phi = 0.20]. Comorbid PTSD and chronic pain at clinical levels was found in 35 outpatients (57%).

No significant difference in pain locations was found in outpatients with and without current PTSD diagnosis. Further, no significant difference was found between outpatients with PTSD diagnosis (M = 4.74; SD = 2.1) and those without it (M = 3.82; SD = 2.2) [t(59) = −1.33, p = 0.19, adjusted p = 1.000] in the total number of chronic pain locations. The mean difference was = −0.92, 95% CI:-2.31 to 0.46, and the magnitude was small to moderate (eta squared = 0.03). Frequencies and p-values are presented in Table 5

**Table 5:** **Differences between groups on pain locations and total number of chronic pains**

	No PTSD (n = 11)		PTSD (n = 50)		Chi^2^/t-value	p-value	p-value§
Variables	N	%	N	%			
Chronic pain locations*							
Stomach pain	5	14.3	30	87.7	0.780	0.504	1.000
Chest pain	7	15.6	38	84.4	0.712	0.457	1.000
Arms/legs pain	6	15.0	34	85.0	0.723	0.483	1.000
Back pain	6	15.8	32	82.2	0.343	0.733	1.000
Joints pain	6	18.2	27	81.8	0.001	1.000	1.000
Head pain	7	14.3	42	85.7	2.366	0.203	1.000
Pain during urination	2	10.0	18	90.0	1.299	0.312	1.000
Other pain locations	3	15.8	16	84.2	0.094	1.000	1.000
Chronic pain at clinical levels	5	12.5	35	87.5	2.406	0.164	-
Total number of pain locations # (Mean (SD)	3.82 (2.2)		4.74 (2.1)		−1.331	0.190	-

Exact chi-square tests were performed; (*) Chronic pain at clinical levels; (#) T-tests; (§) p-values adjusted for multiple testing by Holm adjustment.

## Discussion

### Chronic pain and traumatic exposures

In our study of 61 psychiatric outpatients, forty (65.6%) reported chronic pain at clinical levels. This is three and a half times higher than rates of chronic pain found in the general population (19%) (Breivik et al. 2006), similar to rates found in other investigations of refugee populations (Jamil et al. 2006; Cheung 1994), and even still higher than a study of pain in refugees resettled in Sweden (Hermansson et al. 2001). Further, we found a mean number of 4.6 chronic pain locations in our outpatients, which is comparable with the number of body parts with pain reported after migration in a previous study (Carlsson et al. 2005), and higher than a study from the Netherlands which found a mean of 2 chronic conditions (Gerritsen et al. 2006). Other studies among Bhutanese, Bosnian and Kosovar refugees exposed to torture found higher number of somatic complains than in our study (van Ommeren et al. 2002; Schubert and Punamäki 2010. The most frequent chronic pain location in our sample was in the head, which is in line with findings from other studies of refugee populations (Moisander and Edston 2003; Moore and Boehnlein 1991). An increase in pain symptoms (pain in the head, pain in the back and pain in the feet) over a ten year period was found in a study of tortured refugees resettled in Denmark (Olsen et al. 2007). It seems that in the case of pain, the passage of time does not heal all wounds, but rather can aggravate them.

We found no significant differences in exposure to specific types of traumatic events between outpatients with chronic pain at clinical levels and those without. One possible explanation can be that all outpatients were exposed to multiple types of traumatic events, thus making the two groups less distinct from each other. It may be difficult to compare only one type of traumatic exposure in refugees, given as a rule rather than as an exception, that they are usually exposed to multiple traumatic events during the pre- and trans-migration journey (Jamil et al. 2010; Pumariega et al. 2005). Further, the heterogeneity of the traumatic exposures seen in our patients may give rise to different pains. A Danish study of torture survivors found a relationship between the four types of torture (Palestinian hanging, falanga, beating and kicking of the head, and positional torture) and specific neuropathic pain conditions (Thomsen et al. 2000). Further, Leeuw and his colleagues found a difference in PTSD symptom severity between pain patients with either muscle tension pain or with headache pain, with the former group reporting higher rates of PTSD symptoms (Leeuw et al. 2007).

### Chronic pain and psychiatric symptomatology

We found that outpatients with chronic pain at clinical levels had significantly more posttraumatic symptoms, although this result is barely significant after adjustment for multiple testing. They also had more psychiatric morbidity than outpatients without chronic pain, also after adjustment for multiple testing. The finding supports our first hypothesis, and is in line with other studies that found that chronic pain often accompanies mental disorders, influencing an increase in their severity (Lepine and Briley 2004; Ohayon 2004). Further, pain has been found to increase the level of psychological distress, especially depression and anxiety symptoms (Tsang et al. 2008). We also found that chronic pain at clinical levels was comorbid with PTSD, and the comorbidity was high (57%) as compared with a study of Bosnian patients with PTSD (Avdibegovic et al. 2010), but lower than in a clinical sample of refugees with different national origins in Norway (Dahl et al. 2006) and Iraqi Gulf veterans refugees in USA (Jamil et al. 2006).

An explanation for the comorbidity between chronic pain and PTSD, as well as for a possible increase in the severity of PTSD symptomatology through the presence of pain, may be through the mutual maintenance mechanism.

The part of the second hypothesis that women would report significantly higher frequencies of chronic pain locations than men in our study was confirmed, which is in line with the mainstream research on gender differences in chronic pain prevalence (Unruh 1996; Tsang et al. 2008; Schubert and Punamäki 2010; Celentano et al. 1990). In contrast, a study on tortured refugees found no significant gender differences (Williams et al. 2010). The other parts of the second hypothesis that women would have more chronic pain at clinical levels and more PTSD and chronic pain comorbidity were not supported by our findings. The lack of gender differences on these items may be because the men participating in our sample also reported high levels of pain at the clinical level, such that the difference between men and women was minimized, and the limited size of our sample did not provide enough power to detect the gender difference reported elsewhere in the literature.

Our third hypothesis was not confirmed; we found no significant difference between patients with and without PTSD in the clinical level of chronic pain in any of the 8 pain locations. We were expecting that the presence of a PTSD diagnosis would increase the prevalence of pain in any of the 8 locations where we measured the presence of chronic pain, but we found no evidence for this. One possible explanation may be that both groups have high rates of comorbidity with other psychiatric diagnoses, making them more similar to each other and possibly resulting in no significant difference in chronic pain at clinical levels.

Regarding clinical implications, the present study indicates that chronic pain should be assessed and addressed in the treatment of multi-traumatized refugees in outpatient psychiatric clinics, in addition to mental health problems, knowing that chronic pain can increase the duration and the severity of mental disorders (Villano et al. 2007; Carlsson et al. 2010).

Chronic pain and PTSD are two distinct disorders and they often occur together, and due to their mutual maintenance there is a need for assessment and treatment for both conditions in outpatients with a refugee background. The outpatients need to be made more aware about the intersection of chronic pain with PTSD. Improved awareness of the internal and external cues of both chronic pain and PTSD symptoms will help patients achieve more positive coping through both cognitive and behavioral strategies, and to break the vicious circle of mutual maintenance of chronic pain and PTSD.

The management of comorbid chronic pain and PTSD is still challenging due to the need for clinical evidence-based therapies from longitudinal studies, but the field is developing fast such that new combined therapies for both PTSD and chronic pain are being tested (Liedl et al. 2010).

Future research is needed in order to address more adequately the prevalence rates of chronic pain and comorbid chronic pain and PTSD in refugee populations resettled in a western country using longitudinal designs with control groups from the same ethnic group. Future research may also assess more chronic pain locations, a larger spectrum of stressors and trauma types, including losses, during pre-, trans and post-migration periods, more diagnoses including Axis II personality disorders, as well as socioeconomic and cultural factors.

### Strengths

One strength of this study is that we used a clinical structured interview for assessing PTSD diagnosis. We found 100% agreement between the clinical structured interview (SCID-PTSD) and IES-R in assessing PTSD diagnosis, thus ensuring the validity of the PTSD diagnosis. The validity of the psychiatric diagnoses in refugee populations has been questioned in a number of articles (Hollifield et al. 2002; Al-Saffar et al. 2004), but the general view is that the traumatic reactions described in the diagnostic criteria of PTSD are consistent across cultures (Carlson and Rosser et al.^1994^). However, some specific reactions may be seen in certain cultures (Hinton and Lewis-Fernandez 2011). Another strength was the large diversity of our population, a diversity seen in many clinical settings in Norway (Fosse and Dersyd 2007). A further strength of this study was that we assessed the comorbidity between PTSD and chronic pain at the same time, using validated clinical instruments, ensuring that the PTSD diagnosis and the chronic pain condition were valid.

### Limitations

Our study also has some methodological limitations. The comparisons of our study with other studies should be interpreted with caution, knowing that these comparisons are much influenced by the use of different instruments for assessing chronic pain, and in addition to this, they may assess different number of chronic pains. First, our sample size was relatively small and we used a cross-sectional design without a control group which may limit the generalizability of the findings. Second, we did not use a physician’s medical diagnosis or a widely used clinical instrument for assessing chronic pain locations and intensity, but instead a scale from one clinical interview created for assessing the DESNOS syndrome. This may limit our findings and conclusions with regard to the total number of chronic pain locations and chronic pain intensity, and the results should thus be interpreted with caution. Third, we did not assess when the chronic pain began to see if it was related to the development of the PTSD diagnosis, thus we do not know if the chronic pain was a result of the development of a PTSD diagnosis, or if these two are unrelated. Future research needs to assess more clearly the onset of chronic pain and how it is temporally related to a PTSD diagnosis. Fourth, our exclusion of outpatients with a lack of proficiency in the Norwegian language may have excluded many participants who would have otherwise been eligible, thus the prevalence rates may be biased and should be interpreted with caution. Fifth, the timeframe to complete and send back the Questionnaire was agreed to be one week after the clinical interview, but no clear date was chosen for the completion of several self-reported questionnaires like IES-R and HSCL-25 which assess the symptoms from the last 7 days. We are not sure if all the outpatients chose the same date to complete the self-reported questionnaires. Sixth, we have assessed some post-migration-living difficulties, but not all difficulties that resettled refugees in a Western country may encounter. There is evidence that the current living difficulties have a deep impact on the refugees and many acknowledge these difficulties as having a stronger impact on psychological health than previous exposure to traumatic events in the country of origin. Lastly, the majority of our sample reported multiple pain sites, and thus we do not know which pain site contributed more to the overall pain disability and intensity.

## Conclusions

In our study we found high rates of PTSD and chronic pain, with a majority of outpatients reporting comorbidity between the two. Only a few studies in refugee populations have measured this comorbidity and found such a high level of PTSD and chronic pain at clinical levels. This comorbidity is related to increased severity of psychiatric symptoms and high psychiatric morbidity, possibly due to a mutual maintenance mechanism between the two conditions. Because of the mutual influence between the two disorders, it is important that they be assessed and treated together. Chronic pain should be acknowledged as an independent clinical entity, just in the same way as PTSD is acknowledged as a distinct psychiatric disorder. Further investigations into the comorbidity of PTSD with other somatic disorders, as well as the comorbidity of chronic pain with other psychiatric conditions, would help to identify factors associated with these comorbidities. Future studies should also include validated scales for post-migration-living difficulties which have been found to have a strong impact on the resettled refugees’ mental health and quality of life.
